# ARP2/3 complex is required for directional migration of neural stem cell-derived oligodendrocyte precursors in electric fields

**DOI:** 10.1186/s13287-015-0042-0

**Published:** 2015-03-21

**Authors:** Yongchao Li, Pei-Shan Wang, George Lucas, Rong Li, Li Yao

**Affiliations:** Department of Biological Sciences, Wichita State University, Fairmount 1845, Wichita, KS 67260 USA; Stowers Institute for Medical Research, 1000 E 50th Street, Kansas City, MO 64110 USA; Department of Orthopaedics, Via Christi Hospital, 929 St Francis North, Wichita, KS 67214 USA; Department of Orthopaedics, School of Medicine-Wichita, University of Kansas Medical Center, 1010 N Kansas Street, Wichita, KS 67214 USA; Department of Medical Sciences, School of Medicine-Wichita, University of Kansas Medical Center, 1010 N Kansas Street, Wichita, KS 67214 USA

## Abstract

**Introduction:**

The loss of oligodendrocytes in a lesion of the central nervous system causes demyelination and therefore impairs axon function and survival. Transplantation of neural stem cell-derived oligodendrocyte precursor cells (NSC-OPCs) results in increased oligodendrocyte formation and enhanced remyelination. The directional migration of grafted cells to the target can promote the establishment of functional reconnection and myelination in the process of neural regeneration. Endogenous electric fields (EFs) that were detected in the development of the central nervous system can regulate cell migration.

**Methods:**

NSCs were isolated from the brains of ARPC2^+/+^ and ARPC2^−/−^ mouse embryo and differentiated into OPCs. After differentiation, the cultured oligospheres were stimulated with EFs (50, 100, or 200 mV/mm). The migration of OPCs from oligospheres was recorded using time-lapse microscopy. The cell migration directedness and speed were analyzed and quantified.

**Results:**

In this study, we found that NSC-OPCs migrated toward the cathode pole in EFs. The directedness and displacement of cathodal migration increased significantly when the EF strength increased from 50 to 200 mV/mm. However, the EF did not significantly change the cell migration speed. We also showed that the migration speed of ARPC2^−/−^ OPCs, deficient in the actin-related proteins 2 and 3 (ARP2/3) complex, was significantly lower than that of wild type of OPCs. ARPC2^−/−^ OPCs migrated randomly in EFs.

**Conclusions:**

The migration direction of NSC-OPCs can be controlled by EFs. The function of the ARP complex is required for the cathodal migration of NSC-OPCs in EFs. EF-guided cell migration is an effective model to understanding the intracellular signaling pathway in the regulation of cell migration directness and motility.

**Electronic supplementary material:**

The online version of this article (doi:10.1186/s13287-015-0042-0) contains supplementary material, which is available to authorized users.

## Introduction

The loss of oligodendrocytes in a lesion of the central nervous system (CNS) causes demyelination and therefore impairs axon function and survival. Transplantation of oligodendrocyte precursor cells (OPCs) results in increased oligodendrocyte formation and enhanced remyelination. Cell motility is an important functional property of neural stem cells (NSCs). Effectively directed migration of grafted NSC-derived OPCs (NSC-OPCs) to the target can promote the establishment of functional reconnection and myelination after injury or disease. Physiological electric fields (EFs) play an important role in the development of the CNS [1-3]. The application of EFs *in vivo* enhanced the regrowth of damaged spinal cord axons with some success [4]. *In vitro* studies have shown that EFs can direct spinal neuron axon growth toward the cathode [5,6] and guide the migration of various types of cells [7-12].

Recent studies have shown that primary neural cells, some types of stem cells, and stem cell-derived neurons can respond to EFs and display directional migration [13-18]. However, the influence of EFs on the migration direction of these cells was variable. Hippocampal neurons migrated to the cathode [13], whereas chicken Schwann cells migrated to the anode in EFs [19]. The embryonic and adult neural progenitor cells migrated to the cathode pole in an applied EF [14]. NSCs derived from human embryonic stem cells (hESCs) migrated to the cathode [15]. We recently reported that both the differentiated NSCs from embryoid bodies and embryonic stem cell-derived motor neurons can be guided to migrate toward the cathode in EFs [17]. Bone marrow mesenchymal stromal cells (BM-MSCs) migrated to the cathode in EFs. The EF threshold that induced directional migration of BM-MSCs was about 25 mV/mm [18]. Human induced pluripotent stem cells (iPSCs) migrated to the anode pole in EFs, whereas hESCs migrated toward the cathode [16]. These research outcomes indicate that EFs may direct transplanted or endogenously regenerating OPCs to migrate to a lesion in the CNS to remyelinate regenerated axons.

The leading edge of a migrating cell guides its direction. Polymerization of actin filaments underneath the plasma membrane is the main driving force for protrusions on the leading edge. One of the evolutionarily conserved regulators of actin nucleation is the actin-related proteins 2 and 3 (ARP2/3) complex [20,21]. The ARP2/3 complex concentrates at the leading edges and nucleates new actin filaments to form branches from preexisting filaments, therefore driving the lamellipodia protrusion. The main activators of the ARP2/3 complex are the Wiskott-Aldrich syndrome protein (WASP) and the suppressor of the cyclic-AMP receptor (SCAR) mutation together with the WASP and verprolin (WAVE) homologous protein or SCAR/WAVE. These proteins mediate the function of ARP2/3 for actin filament branching and growth. Previous studies have demonstrated the critical role of ARP2/3 in the generation of protrusive actin structures and cell motility. The downregulation of ARP2/3 components or dominant-negative constructs derived from WASP family proteins inhibited lamellipodia formation or morphology [22-24]. Cells failed to form stereotypical lamellipodia or undergo sustained directional migration after the ARP2/3 complex was genetically disrupted [25]. It was reported that the migration of OPCs involves dynamic morphological changes driven by actin cytoskeletal rearrangements [26]. However, the function of the ARP2/3 complex in EF-directed cell migration has not been reported previously, and studying its role in the navigation of NSC-OPCs will help to explore the mechanism of EF-guided cell migration.

In this study, mouse NSCs were differentiated into OPCs, and the migration of NSC-OPCs in an applied EF was characterized. We determined the migration direction and velocity of NSC-OPCs in EFs as well as the relationship of EF strength and OPC migration directness and motility. The ARP2/3 complex was genetically disrupted in the cells in order to determine its function in cell motility and directedness. We derived mouse NSC lines from ARPC2 floxed allele mouse embryos where ARPC2 was conditionally deleted by nestin-Cre. We studied the migration of ARPC2^−/−^ NSC-OPCs in EFs to demonstrate the critical role of the ARP2/3 complex in EF-directed cell migration.

## Methods

### ARPC2^+/+^ and ARPC2^−/−^ neural stem cell derivation and differentiation into oligodendrocyte precursor cells

All procedures of the animal work in this study were approved by the Institutional Animal Care and Use Committee of the Stowers Institute of Medical Research. E14.5-E17.5 mouse cortices were isolated in Hank’s Balanced Salt Solution (Life Technologies, Grand Island, NY, USA) on ice. After removal of the meninges, the brain was cut into small pieces and centrifuged at 600 revolutions per minute for 5 minutes. The resulting pellet was mixed with an ice-cold neural culture medium (NCM)—Dulbecco’s modified Eagle’s medium: nutrient mixture F-12 (DMEM/F12) medium supplemented with N2, B27 (Life Technologies)—and 10 μg/mL insulin (Sigma-Aldrich, St. Louis, MO, USA). This brain tissue was triturated with a 1-mL pipet tip and passed through a 40-μm cell strainer. The dissociated cells were cultured in a neurosphere growth medium—NCM supplemented with 20 ng/mL basic fibroblast growth factor (bFGF) and 20 ng/mL epidermal growth factor; Peprotech, Rocky Hill, NJ, USA—for 3 to 4 days to form neurospheres. The cells were passaged every 3 to 4 days. Neurospheres were dissociated by Accutase (Life Technologies). Then the cells were cultured in oligosphere medium—NCM supplemented with 20 ng/mL bFGF and 20 ng/mL platelet-derived growth factor (PDGF-AA) (Peprotech)—for 5 to 6 days to form oligospheres. The oligospheres were passaged every 5 to 6 days. After the cells were passaged at least three times, the oligospheres were used to investigate the migration of oligodendrocyte precursors in EFs. For differentiation of the NSC-OPCs to oligodendrocytes, the oligospheres were cultured in the poly-DL-ornithine (100 μg/mL) and laminin (20 mg/mL; Sigma-Aldrich) coated wells for 24 to 48 hours to allow the NSC-OPCs to migrate out of the spheres. Then the cell culture medium was changed to NCM with the supplement of Tri-iodothyronine (T3) and N-acetylcysteine (NAC), and the cells were cultured for 7 days.

Nervous system-specific conditional knockout ARP2 mouse embryos (Arpc2^lox/lox^nestin-Cre) were generated by mating mice carrying an ARPC2 allele flanked by loxP sites with mice carrying an ARPC2 allele flanked by loxP sites and nestin-Cre. ARPC2^+/+^ mouse embryos carry wild-type alleles and nestin-Cre. In this study, NSCs were isolated from the brains of ARPC2^+/+^ and ARPC2^−/−^ mouse embryo and differentiated into OPCs by using the method described above.

### Migration of neural stem cell-derived oligodendrocyte precursor cells in electric fields and time-lapse imaging

To investigate the migration of NSC-OPCs in EFs, EFs were applied to the cultured cells as reported previously [13,27]. In brief, the differentiated oligospheres were grown in a glass chamber made of coverslips. The final dimensions of the chambers were 30 × 0.8 × 0.15 mm. The cell culture surface of the chamber was coated with poly-DL-ornithine (100 μg/mL) and laminin (20 mg/mL). To apply the EFs to the cultured cells in the chamber, agar-salt bridges—filled with Steinberg’s solution (58 mM NaCl, 0.67 mM KCl, 0.44 mM Ca(NO_3_)_2_ · 4H_2_0, 1.3 mM MgSO_4_ · 7H_2_0, and 4.6 mM Trizma base gelled with 1% agar—were used to connect silver-silver chloride electrodes in beakers of Steinberg’s solution and pools of cell culture medium at either side of the chamber. Culture conditions in the control were identical except that no EFs were applied.

To study the migration of NSC-OPCs from oligospheres in an applied EF, the oligospheres were placed in the chamber and cultured with oligosphere medium. After being cultured for 1 hour, the oligospheres attached to the plastic surface of the chamber, and some cells migrated out of the oligospheres. Then steady direct current (DC) EFs of 200 mV/mm were applied to the cultured oligospheres for 8 hours in a CO_2_ incubator (37°C). Voltage was supplied with a Bio-Rad 200/2.0 Power Supply (Bio-Rad Laboratories, Hercules, CA, USA). The EFs were measured with an electric meter (DT830B, Jameco Benchpro; Jameco Electronics, Belmont, CA, USA). At the beginning and end of the stimulation, the voltage was measured to check the constancy of the applied EFs. Then the oligospheres were fixed with 4% paraformaldehyde.

To track the individual cell migration, the oligospheres were seeded in the glass chamber for 10 to 18 hours. Then steady DC EFs of 50, 100, and 200 mV/mm were applied to the cultured cells in the chambers. The cell migration from the oligospheres with or without EF stimulation was recorded by time-lapse microscopy. The microscope (Zeiss Axio Observer microscope; Carl Zeiss, Oberkochen, Germany) was placed in a plastic incubator at 37°C and with 5% CO_2_. Sterile conditions were maintained throughout. To study the migration of cells in an applied EF, an area with cells was selected and the cell migration was recorded. Time-lapse imaging was performed by using ZEN 2011 imaging microscope software (Carl Zeiss), and the images were recorded by digital camera (AxioCam MRm Rev.3 with FireWire; Carl Zeiss). Cell migration was recorded by capturing images every 5 minutes during a 2-hour period. To confirm cell migration direction in the EFs, the cell migration from oligospheres was recorded for 2 hours in the EF (200 mV/mm); then the EF polarity was reversed without changing the field strength, and cell migration was recorded for 2 hours.

### Quantification of cell migration

The time-lapse images were analyzed by ImageJ software (National Institutes of Health, Bethesda, MD, USA). Cell migration was quantified by using the method reported previously [27,28]. The angle at which each cell moved with respect to the imposed EF direction was measured. The mean directedness of total cell movement was calculated from the equation Σicos θi/n, where θ is the angle between the field vector and the cellular translocation direction, and n is the total number of cells. The cosine of the angle would be equal to 1 if the cell moved directly along the field lines toward the cathode, 0 if the cell moved perpendicularly to the field direction, and −1 if the cell moved directly toward the positive pole of the field. The net displacement was the displacement of the cell migration along the field line. The cell migration velocity was calculated from the full distance of cell migration in a given time. To quantify the cell migration speed, directedness, and displacement, cells from three independent experiments were analyzed.

### Immunocytochemistry

After the EF stimulation, the NSC-OPCs and oligospheres were fixed with 4% paraformaldehyde and permeabilized with 0.2% Triton X-100. Cells were exposed to a blocking solution—10% horse serum and 1% bovine serum albumin (BSA)—for 20 minutes. All antibodies were diluted in phosphate-buffered saline with 1% BSA. The cells were incubated with monoclonal anti-nestin antibody (Millipore, Billerica, MA, USA), polyclonal anti-platelet-derived growth factor receptor alpha (anti-PDGFRα) antibody (Santa Cruz Biotechnology, Inc., Dallas, TX, USA), and monoclonal anti-A2B5 antibody (generated in Q. Richard Lu’s lab, Cincinnati Children’s Medical Center) to determine the cell phenotype. Some cells were labeled with monoclonal primary antibody anti-p34-Arc/ARPC2 (Millipore) and rhodamine phalloidin (Life Technologies) to localize the ARP2/3 complex. The differentiated NSC-OPCs were labeled with monoclonal anti-O4 antibody (generated in Q. Richard Lu’s lab). The secondary antibodies were Alexa Fluor® 594 donkey anti-goat IgG and Alexa Fluor® 488 donkey anti-mouse IgG (Jackson ImmunoResearch Laboratories, Inc., West Grove, PA, USA). Nuclei were stained with diamidino-2-phenylindole (DAPI).

### Statistics

Statistical analysis was performed by using the two-tailed Student’s *t* test, where data are expressed as the mean ± standard deviation. Statistical significance was placed at a *P* value of less than 0.05.

## Results

### Neural stem cell-derived oligodendrocyte precursor cells migrated toward cathode in an applied electric field

**Figure 1:**
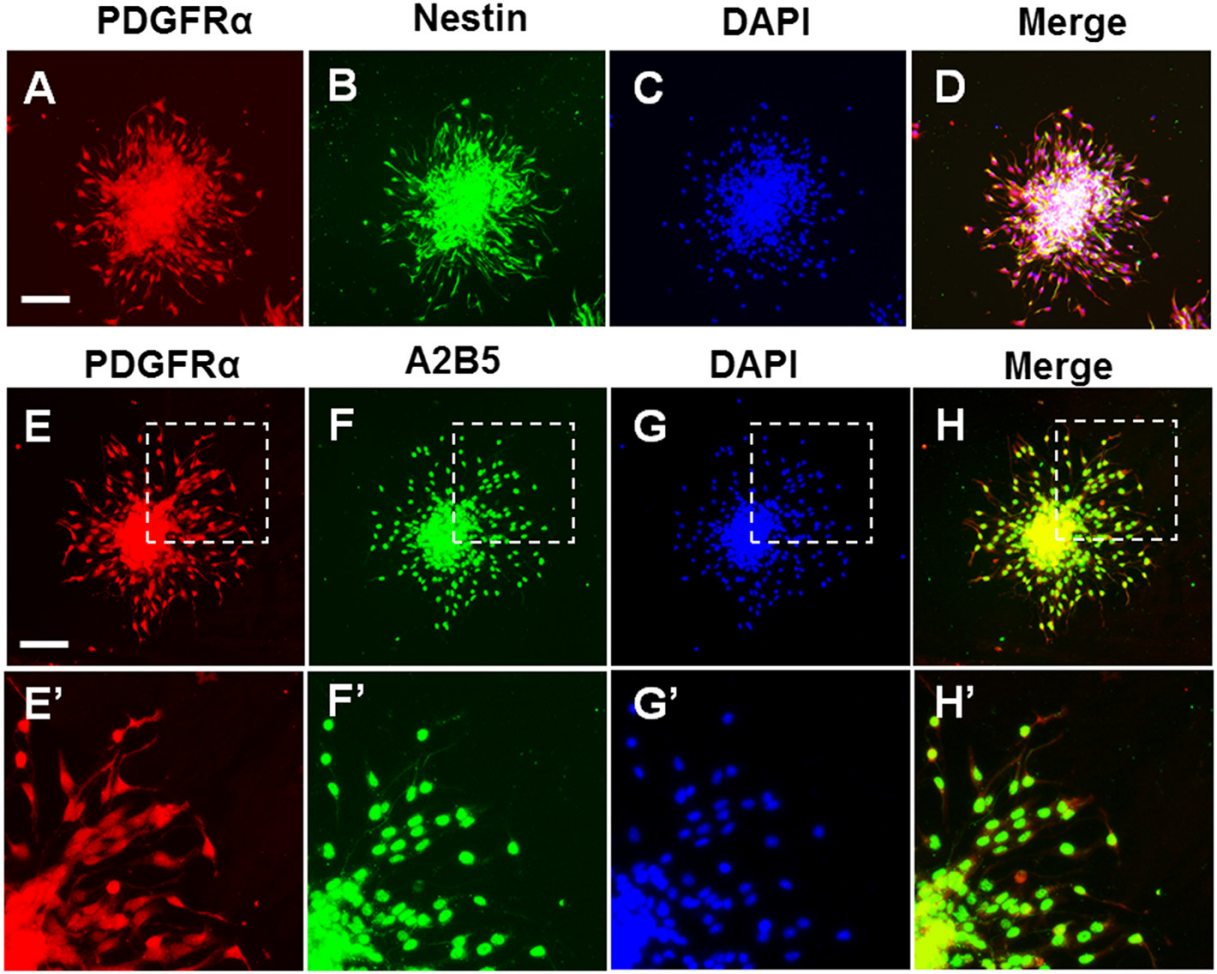
**Differentiation of NSC-OPCs.** The cells that migrated out of the oligospheres were labeled with antibodies and DAPI. **(A)** Cell staining with anti-PDGFRα antibody (red); **(B)** Cell staining with anti-nestin antibody (green); **(C)** Nuclei staining with DAPI, **(D)** Merged image of images **(A)**–**(C)**, **(E)** Cell staining with anti-nestin antibody (green), **(F)** Cell staining with anti-A2B5 antibody (green), **(G)** Nuclei staining with DAPI, **(H)** Merged image of images **(E)**–**(G)**. **(E’–H’)** Magnified images of inset indicated in **(E–H)**, respectively. Scale bar: 100μm.

After oligospheres were cultured in the cell culture plate for 10 hours, some cells migrated out and distributed symmetrically around the oligospheres. To verify the phenotype of the cells, PDGFRα, A2B5, and nestin antibodies were used to label the cells. Cells that migrated out of the oligospheres showed the positive phenotype of the NSC and oligodendrocyte precursors (Figure 1A-H′).

**Figure 2:**
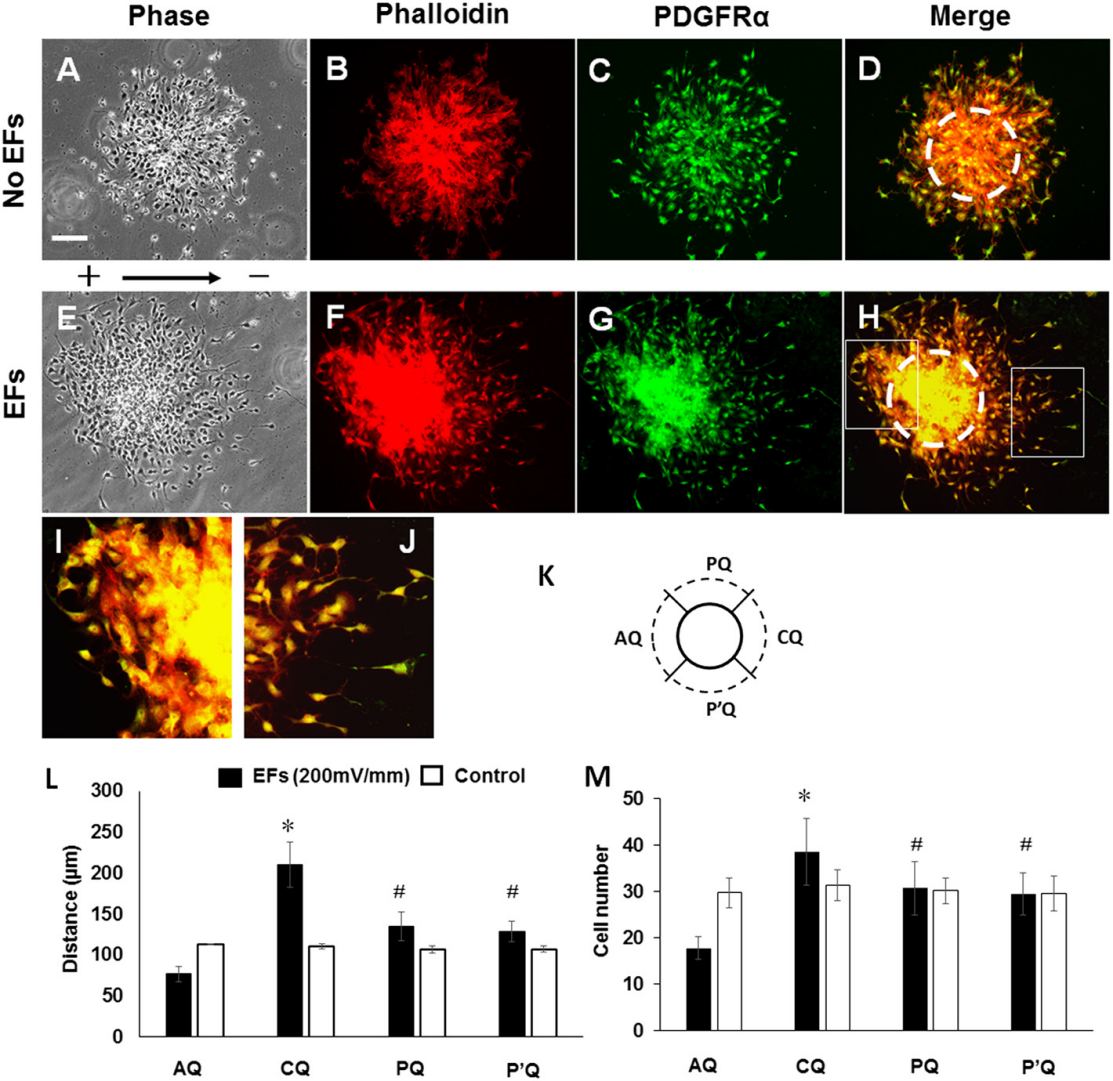
**Analysis of cell migration from oligospheres. (A-D)** Symmetrical distribution of ARPC2^+/+^ neural stem cell-derived oligodendrocyte precursor cells (NSC-OPCs) around oligosphere without electric field (EF) stimulation. **(E-H)** Asymmetrical distribution of NSC-OPCs around oligosphere exposed to EF for 8 hours. Dashed cycle lines in **(D)** and **(H)** show core area of oligospheres. **(B, F)** Cells labeled with rhodamine phalloidin (red). **(C, G)** Cells labeled with anti-platelet-derived growth factor receptor alpha (anti-PDGFRα) antibody (green). **(D)** Overlaid images of **(B)** and **(C)**. **(H)** Overlaid images of **(F)** and **(G)**. Scale bar: 100 μm. **(I, J)** Magnified images of inset indicated in **(D)** and **(H)**, respectively. **(K)** Diagram showing division of quadrants around oligosphere. CQ is cathode-facing quadrant. AQ is anode-facing quadrant. PQ and P′Q are quadrants perpendicular to field line of EFs. Solid circle line represents edge of oligosphere core. Dashed circle line represents frontier of migrated cells. **(L)** Analysis of cell migration distance from oligospheres. **(M)** Analysis of the number of migrated cells from oligospheres. *P <0.01, compared with anode-facing quadrant and quadrants perpendicular to field line of EFs. ^#^ *P* <0.01, compared with anode-facing quadrant.

To study the migration of NSC-OPCs from oligospheres, the cultured oligospheres in some chambers were subjected to EF stimulation (200 mV/mm) for 8 hours. The control oligospheres were cultured without EF stimulation. The immunostaining for OPCs that migrated out of the oligospheres showed that the cells were PDGFRα-positive. In control studies, the cells were symmetrically distributed around the oligospheres (Figure 2A-D). For oligospheres that were stimulated with an EF, the area occupied by the migrated cells was asymmetrical. More cells migrated out from the cathode-facing side of the oligospheres than the anode-facing side (Figure 2E-H).

To quantify the distribution of migrated cells in an EF, the area surrounding each oligosphere was divided into four quadrants (Figure 2K). Quadrant C (CQ) was the cathode-facing quadrant, and quadrant A (AQ) was the anode-facing quadrant. The distance between the oligosphere core and the frontier of migrated cells in each quadrant was measured. In either the control group or the EF-stimulated group, the oligospheres from three independent experiments were quantified. The cells were labeled with the anti-nestin antibody and rhodamine phalloidin to show their morphology.

In the EF stimulation group, the average migration distance of the cells in the cathode-facing quadrant was 210.9 ± 27.7 μm, which is significantly higher than that of the anode-facing side (76.9 ± 6.5 μm, *P* <0.01) and that of the corresponding side (CQ) of the control group (110.9 ± 2.9 μm, *P* <0.01). In the control group, the average cell migration distance in the right (cathode-facing side in EF study) quadrant was not different from the left (anode-facing side in EF study) quadrant (Figure 2L). More cells migrated out of the oligospheres from the cathode-facing side (38.5 ± 7.2) than the anode-facing side (17.8 ± 2.4) (Figure 2M). This suggests that the EF stimulation promoted the polarized cell migration from oligospheres toward the cathode.

### Reversal of electric field poles reversing the migration direction of neural stem cell-derived oligodendrocyte precursor cells in electric fields

**Figure 3:**
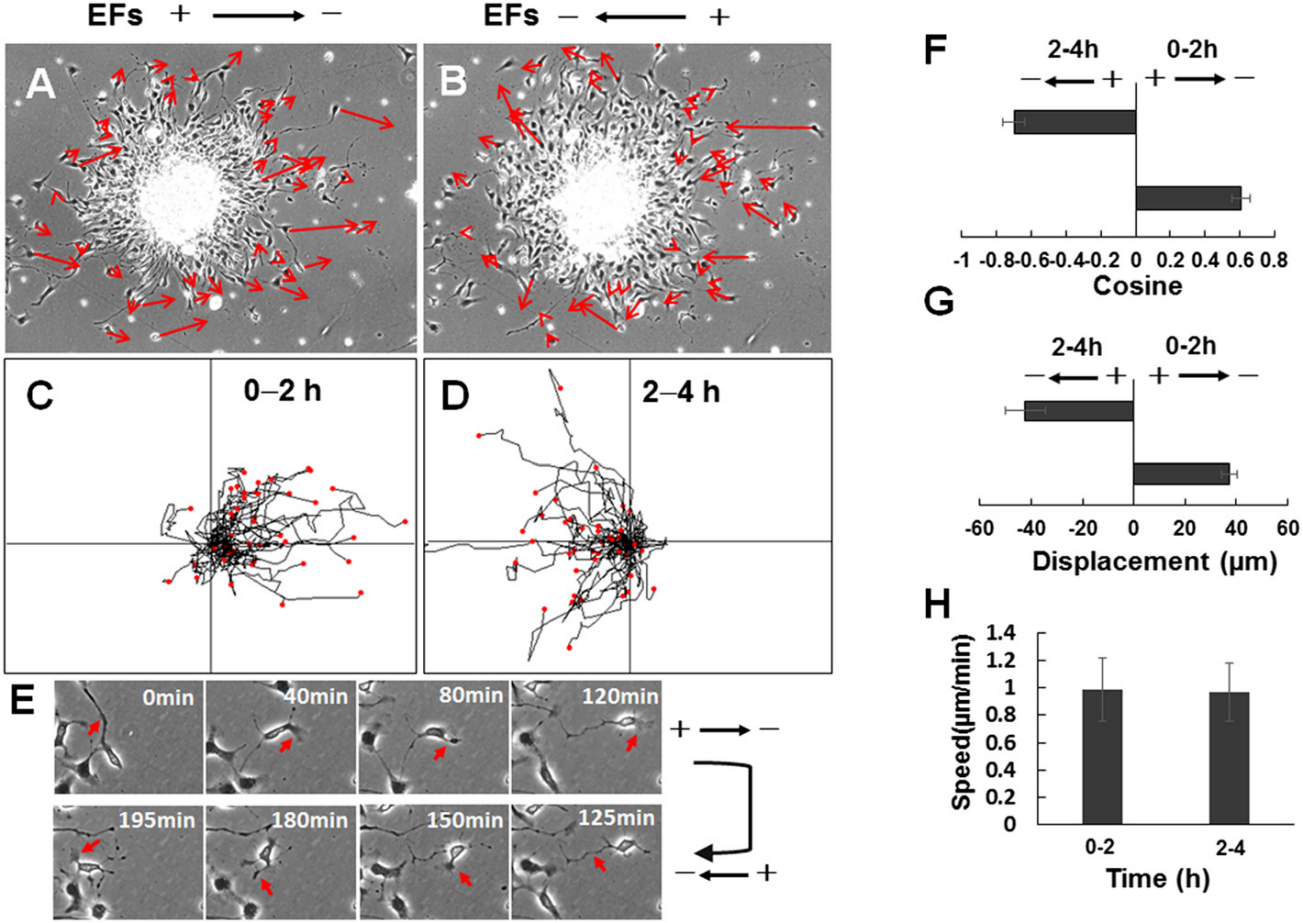
**Reversal of migration direction of ARPC2** ^**+/+**^ **neural stem cell-derived oligodendrocyte precursor cells with reversal of electric field (EF) vectors. (A)** Cell migration to cathode pole from oligosphere in EF of 200 mV/mm. **(B)** Reversed migration of same cells in EF of 200 mV/mm. Arrows show migration direction and positions of cells at beginning and end of each experiment. Scale bar: 100 μm. **(C)** Tracks of cathodal migration of cells in **(A)** in EFs. **(D)** Migration tracks of cells in **(B)** after EF is reversed. Cells switched direction to migrate to new cathode. **(E)** Migration of representative cell in EFs. The leading process of the cell guides cell migration toward the cathode during the first 2 hours. Cell migration in opposite direction when EF pole is reversed. **(F)** Reversal of directedness and **(G)** reversal of net displacement of cell migration when EF pole is switched to opposite direction. **(H)** No significant change in cell migration rates before and after EF pole reversal. See Additional file 1: Video 1.

In the 2-hour recording period, NSC-OPCs migrated to the cathode pole in the EF of 200 mV/mm (Figure 3A). To confirm the response of OPCs to the EF application, EF polarity was reversed, and the migration was recorded for 2 hours. After reversal of the field polarity, cells migrated toward the new cathode (Figure 3B and Additional file 1: Video 1). The tracking of cell migration and the analysis of individual cell migration clearly showed reversal of the cell migration path (Figure 3C-E). The magnitudes of directedness of the cells before and after reversal of the EF poles were 0.61 ± 0.05 and −0.70 ± 0.06, respectively (Figure 3F). The net displacements of the cells before and after reversal of the EF poles were 37.31 ± 3.2 and −42.33 ± 8.02 μm, respectively (Figure 3G). This reversal of migration direction again indicates that migration was directed by EFs. However, before EF poles were reversed, the migration speed was 0.99 ± 0.23 μm/minute, which was not significantly different from the speed of 0.98 ± 0.21 μm/minute, after the EF poles were reversed (Figure 3H).

### ARPC2^−/−^ neural stem cell-derived oligodendrocyte precursor cells migrated and oriented randomly in electric fields

**Figure 4:**
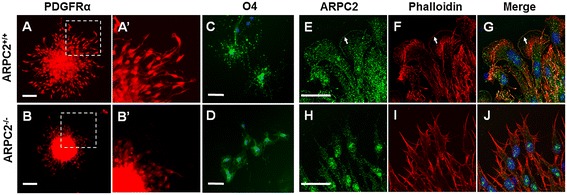
**Localization of ARPC2 and actin in neural stem cell-derived oligodendrocyte precursor cells (NSC-OPCs). (A)** Migration of ARPC2^+/+^ NSC-OPCs out of oligosphere. **(B)** Migration of ARPC2^−/−^ NSC-OPCs out of oligosphere. **(A, B)** Cells labeled with anti-platelet-derived growth factor receptor alpha (anti-PDGFRα) antibody (red). **(A′, B′)** Magnified images of inset indicated in **(A and B)**, respectively Scale bar: 100 μm. **(C)** Differentiation of ARPC2^+/+^ NSC-OPCs to oligodendrocytes. **(D)** Differentiation of ARPC2^+/+^ NSC-OPCs to oligodendrocytes. Scale bar: 50 μm. **(E-G)** ARPC2^+/+^ NSC-OPCs are labeled with anti-ARPC2 antibody. Arrows show labeled ARPC2 at leading edges of the cells. **(H-J)** ARPC2 is absent at leading edge of ARPC2^−/−^ NSC-OPCs. **(E, H)** Cells labeled with anti-ARPC2 antibody (green). **(F, I)** Cells labeled with Alexa Fluor 568 phalloidin (red). Scale bar: 30 μm.

After the oligospheres of ARPC2^−/−^ NSC-OPCs were cultured for 10 hours, fewer cells migrated out the oligospheres than that of oligospheres of ARPC2^+/+^ NSC-OPCs (Figure 4A-B′). The differentiation of ARPC2^+/+^ NSC-OPCs and ARPC2^−/−^ NSC-OPCs was observed after the cells were cultured in NCM with the supplement of T3 and NAC for 7 days. Both ARPC2^+/+^ oligodendrocytes (Figure 4C) and ARPC2^−/−^ oligodendrocytes (Figure 4D) were labeled with anti-O4 antibodies and developed multiple processes. However, the ARPC2^−/−^ oligodendrocytes showed smaller cell size and shorter process compared with ARPC2^+/+^ oligodendrocytes. Alexa Fluor 568 phalloidin staining of cells showed that the ARPC2^+/+^ NSC-OPCs exhibit the typical actin organization with F-actin enriched at the lamellipodia leading edge and stress fibers extending into the lamella (Figure 4F, I). The leading edges of mutant cells are characterized by filopodia-like protrusions that contain actin bundles, followed by prominent transverse actin arcs (Figure 4I). Staining with an anti-ARPC2 antibody confirmed that the ARP2/3 complex localizes to the actin-rich lamellipodia of ARPC2^+/+^ NSC-OPCs and is absent from the tip of the actin bundles in the filopodia-like protrusions of ARPC2^−/−^cells (Figure 4J).

**Figure 5:**
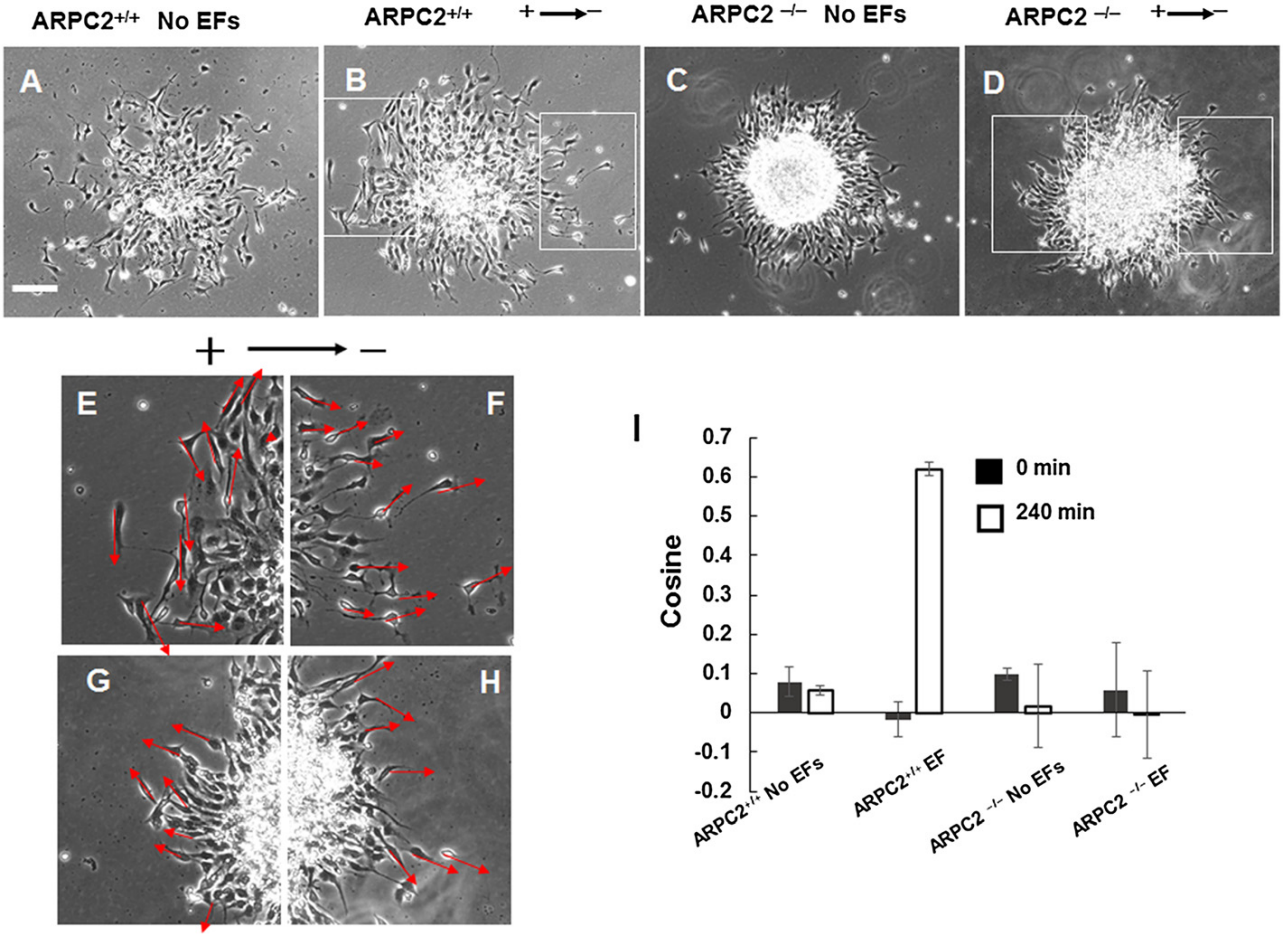
**Orientation of cells in electric fields (EFs). (A)** Random orientation of leading processes of ARPC2^+/+^ neural stem cell-derived oligodendrocyte precursor cells (NSC-OPCs) without EF stimulation. **(B)** Cathode-facing orientation of leading processes of ARPC2^+/+^ NSC-OPCs in EF of 200 mV/mm for 2 hours. **(C, D)** Random orientation of leading processes of ARPC2^−/−^ OPCs without EF stimulation or in EF of 200 mV/mm for 2 hours, respectively. Scale bar: 100 μm. **(E, F)** Magnified images of inset indicated in **(B)**. **(G, H)** Magnified images of insets indicated in **(D)**. Arrows show orientation of leading processes. **(I)** Quantification of leading processes.

The migration of OPCs from oligospheres was recorded for 2 hours with time-lapse microscopy. The orientation of cells before and after the 2-hour recording period was analyzed. Without EF stimulation, the leading processes of ARPC2^+/+^ and ARPC2^−/−^ NSC-OPCs oriented randomly before and after the 2-hour recording time (Figure 5A and C). The direction of the leading processes oriented to face the cathode in the EF of 200 mV/mm) after the 2-hour treatment (Figure 5B). The leading processes of ARPC2^−/−^ NSC-OPCs oriented randomly after 2 hours of EF stimulation (Figure 5D). The quantification of the orientation of cell leading processes showed that the ARPC2^−/−^ NSC-OPCs lost their response to EFs (Figure 5I).

**Figure 6:**
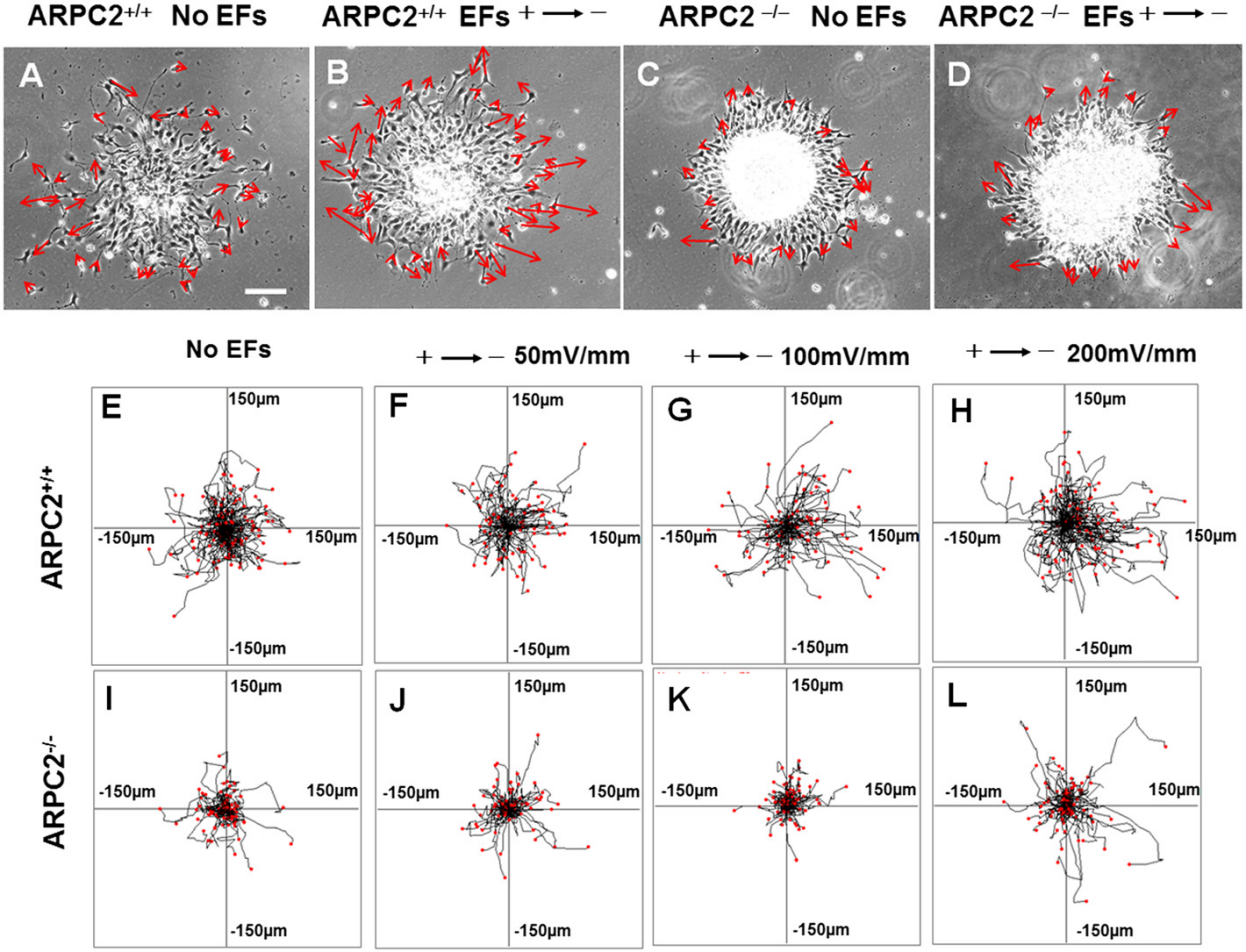
**Migration of neural stem cell-derived oligodendrocyte precursor cells (NSC-OPCs) in electric fields (EFs). (A)** Random migration of ARPC2^+/+^ NSC-OPCs during 2-hour period without EF stimulation. See also Additional file 2: Video 2. **(B)** ARPC2^+/+^ NSC-OPC migration to cathode in EF. See also Additional file 4: Video 4. **(C)** Random migration of ARPC2^−/−^ NSC-OPCs during 2-hour period without EF stimulation. See also Additional file 3: Video 3. **(D)** Random migration of ARPC2^−/−^ OPCs in EF. See also Additional file 5: Video 5. Arrows show migration direction and positions of cells at beginning and end of each experiment. Scale bar: 100 μm. **(E-L)** Migration paths of cells determined by video monitor tracings. Position of all cells at t = 0 minutes is represented by origin position (center of frame) with migratory track of each cell at 120 minutes plotted as single line on graph. Each arm of axes represents 150 μm of translocation distance.

**Figure 7:**
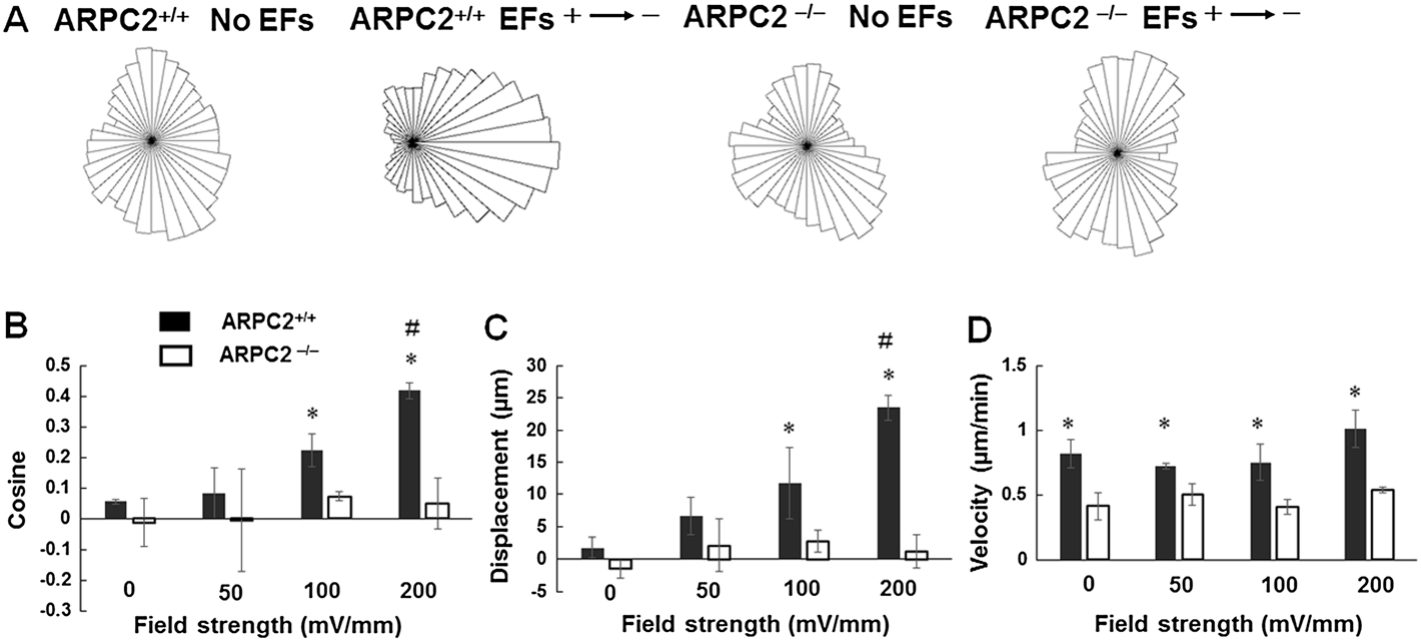
**Directedness, net displacement, and migration speed of neural stem cell-derived oligodendrocyte precursor cell (NSC-OPC) migration in electric fields (EFs). (A)** Circular histogram. The migrated ARPC2^+/+^ NSC-OPCs showed clear biased distribution in EFs (200 mV/mm). It indicated the cathodal migration of the cells. The range of interval is 10 degrees. **(B)** Increase in average cosine (directedness) of ARPC2^+/+^ NSC-OPCs when field strength increased from 50 to 200 mV/mm. No change in average cosine (directedness) of ARPC2^−/−^ NSC-OPCs in EFs from 50 to 200 mV/mm was observed. **(C)** More significant net cathodal displacement of ARPC2^+/+^ NSC-OPCs when EF increased from 50 to 200 mV/mm. No change in net displacement of ARPC2^−/−^ NSC-OPCs when EF increased from 50 to 200 mV/mm was observed. *P <0.05, compared with ARPC2^+/+^ NSC-OPCs without EF stimulation. #<0.05, compared with ARPC2^+/+^ NSC-OPCs in EFs with field strength of 100mM/mm. **(D)** Analysis of cell migration speed in EFs. EFs did not significantly change migration speed of either ARPC2^+/+^ NSC-OPCs or ARPC2^−/−^ NSC-OPCs. *P <0.05, compared with ARPC2^−/−^ NSC-OPCs.

The ARPC2^+/+^ cells (Figure 6A and Additional file 2: Video 2) and ARPC2^−/−^ NSC-OPCs (Figure 6C and Additional file 3: Video 3) migrated randomly from oligospheres without EF stimulation. The ARPC2^+/+^ cells migrated clearly toward the cathode in an applied EF of 200 mV/mm (Figure 6B and Additional file 4: Video 4). However, ARPC2^−/−^ NSC-OPCs showed random migration in the EF of 200 mV/mm (Figure 6D and Additional file 5: Video 5). In Figure 5E-L, each frame shows the superimposed migration tracks of cells. The position of all the cells at t = 0 minutes is represented by the origin (0, 0). Each line represents the migration track of one single cell over a 2-hour period. ARPC2^+/+^ and ARPC2^−/−^ NSC-OPCs that were not stimulated with an EF migrated randomly in the control studies. The ARPC2^+/+^ NSC-OPCs subjected to an applied EF (100 and 200 mV/mm) showed clear cathodal migration. However, ARPC2^−/−^ NSC-OPCs showed random migration in the EFs. Circular histogram (rose diagram) showed the distribution of migration cells. The migrated ARPC2^+/+^ NSC-OPCs showed clear biased distribution in EFs and therefore the graph indicated the cathodal migration of the cells (Figure 7A).

The directedness of EF-directed cell migration showed a voltage-dependent manner. In the EF group, after 2 hours of exposure to EF stimulation of 50 mV/mm, the directedness of ARPC2^+/+^ NSC-OPCs was 0.08 ± 0.01 (total cell number = 79), which was not significantly different from that of random migration in the control group (0.05 ± 0.08, total cell number = 105) (Figure 7B). The directedness of the cells in the EF of 100 and 200 mV/mm was 0.22 ± 0.05 (total cell number = 83) and 0.42 ± 0.05 (total cell number = 105), respectively, which were significantly higher than the directedness in the EF of 50 mV/mm (*P* <0.05). The quantitative data demonstrated that ARPC2^−/−^ NSC-OPCs did not show any directional migration in the EFs. The magnitudes of directedness of ARPC2^−/−^ OPCs in EFs of 50, 100, and 200 mV/mm were −0.01 ± 0.17 (total cell number = 77), 0.07 ± 0.02 (total cell number = 78), and 0.05 ± 0.08 (total cell number = 87), respectively, which are not significantly different from that of control group (−0.01 ± 0.08, *P* <0.05; total cell number = 89).

The net displacement of the ARPC2^+/+^ NSC-OPCs along the field line also showed a cathodal migration pattern. The net displacements of cells in the EFs of 0, 50, 100, and 200 mV/mm were 1.77 ± 1.65, 6.67 ± 2.90, 11.74 ± 5.50, and 21.55 ± 9.70 μm, respectively (Figure 7C). These results demonstrate that the directedness and displacement of cathodal migration increased when the EF strength increased. However, the net displacement of ARPC2^−/−^ NSC-OPCs was not significantly different in the EFs (Figure 7C).

The cell migration velocities for the control group and the groups exposed to EFs were quantified (Figure 7D). We found that EFs did not influence the cell migration speed significantly. The migration speeds of ARPC2^+/+^ cells in EFs of 50, 100, and 200 mV/mm were 0.72 ± 0.03, 0.75 ± 0.14, and 1.01 ± 0.15 µm per minute, respectively, which were not significantly different from the migration speed of the control group (0.81 ± 0.11 µm per minute). We also observed that the migration speeds of ARPC2^−/−^ NSC-OPCs decreased significantly compared with those of ARPC2^+/+^ NSC-OPCs. The migration speeds of ARPC2^−/−^ NSC-OPCs in EFs of 0, 50, 100, and 200 mV/mm were 0.41 ± 0.10, 0.50 ± 0.08, 0.41 ± 0.06, and 0.54 ± 0.02 µm per minute, respectively. The speeds of ARPC2^−/−^ NSC-OPCs were not significantly different in different EF strengths.

## Discussion

In this study, we found an efficient approach to investigate the migration of NSC-OPCs from oligospheres in an applied EF. The cells migrated dynamically from oligospheres without any chemical or mechanical dissociation procedure, and most cells migrated individually after they moved out of the oligospheres. The migration pattern of the cells on the cathode-facing side and non-cathode-facing side was different in the EFs. Similar to the neuron migration from micro-explant and NSC migration from an embryoid body, the NSC-OPCs migrated directly to the cathode pole from the cathode-facing side of the oligospheres, and they oriented their migration direction and migrated to the cathode pole from the other non-cathode-facing side. This orientation in migration clearly demonstrates the guidance effect of EFs on cells. Because most cells from the oligospheres showed cathodal migration, we found that the quantified migration directedness is comparable to the previously reported migration of dissociated neuronal cells in EFs. The threshold of EFs showed that NSC-OPCs were between 50 and 100 mV/mm, which is similar to the migration of hippocampal neurons and ESC-derived neurons in EFs.

During directed cell migration, cells are dynamically polarized in response to an extracellular gradient of signals (for example, chemotaxis and wound healing). Migrating fibroblasts exhibit a polarized cell shape with a membrane ruffling area and filopodia. The leading lamella protrudes forward and forms new attachment sites. Elongation of actin filaments at the leading edge of the cell [29,30] is the main driving force for cell movement. Application of a DC EF produces galvanotaxis in a variety of cultured cells *in vitro*. In galvanotaxis, cells migrate toward the cathode or the anode. Stimulated with EFs, Chinese hamster ovary (CHO) cells elongated and polarized with a distinct leading-edge lamellipodia facing cathodally [31]. Previous experiments showed the asymmetrical distribution of actin at the lamellipodia projecting toward the cathode [32,33]. In this study, we observed the orientation of ARPC2^+/+^ NSC-OPC in EFs. We found that most cells have a clear leading process that guides cell migration. The leading process of cells is oriented toward the cathode pole in EFs. This observation is similar to the orientation of hippocampal neurons of primary culture and ESC-derived motor neurons in EFs in our previous studies [27,34].

The molecules and signaling pathways that control electrotaxis are diverse and include cellular membrane receptors, intracellular signaling molecules, and cytoskeletons. Previous studies have reported the potential effect of signaling pathway molecules on EF-guided migration of stem cells. Rho-kinase (ROCK) inhibitor Y27632 enhanced the viability of stem cells and inhibited EF-guided directional migration in iPSCs and neurons [12]. ROCK inhibition significantly increased the motility of iPSCs and reduced the directionality of iPSCs in an EF [16]. EFs guide the migration of neuronal stem/progenitor cells (NSPCs) toward the cathode. EF-directed NSPC migration requires activation of N-methyl-D-aspartate receptors (NMDARs), which leads to an increased physical association of Rho GTPase Rac1-associated signals to the membrane NMDARs and the intracellular actin cytoskeleton [17]. Actin polymerization is an important aspect of galvanotaxis. Rho family GTPases orchestrate actin filament assembly and disassembly through the control of actin polymerization, branching, and depolymerization. The WASP family members act as scaffolds to integrate signals from small GTPases such as Rac and Cdc42, signal adapters such as Grb2 and Nck, membrane phospholipids such as PIP2, and protein kinases [21]. The chemical gradient or electric signals activate membrane receptors and downstream intracellular signaling elements, and this leads to asymmetrical distribution of the cytoskeleton. The signaling molecules When corneal epithelial cells were exposed to physiological EFs, activation of extracellular signal-regulated kinases (ERKs) and accumulation of F-actin at the leading, cathode-facing side of the cell were shown [33]. The ARP2/3 complex is involved in regulation of actin polymerization and mediates the formation of branched actin networks with an activating nucleation-promoting factor. The ARP2/3 complex may mediate the regulation of upstream molecules such as phosphoinositide 3-kinase (PI3K) and small GTPases on actin function. In this study, we demonstrated that the disruption of the ARP2/3 complex can significantly alter two aspects of the cell migration pattern. First, the migration speed of ARPC2^−/−^ OPCs decreased significantly compared with ARPC2^+/+^ OPCs. Second, ARPC2^−/−^ OPCs lost cathodal orientation and migration function in EFs. These facts suggest that the proper function of the ARPC2/3 complex is critical for cell motility and directionality in electrotaxis. It may function as an important bridge between upstream molecules and actin that steer cell migration direction and control cell motility.

## Conclusions

In this study, we demonstrated that NSC-OPCs migrated toward the cathode pole in EFs. The cathodal migration of NSC-OPCs in EFs showed a voltage-dependent manner. However, EFs did not significantly change the cell migration speed. We also showed that the migration speed of ARPC2^−/−^ NSC-OPCs was significantly lower than that of the wild-type NSC-OPCs. Deletion of ARPC2 in the NSC-OPCs abolished the directional migration of the cells in the EFs. ARPC2^−/−^ NSC-OPCs migrated randomly in EFs with field strengths from 50 to 200 mV/mm. EFs may be developed as a novel strategy for the guided migration of OPCs to myelinate axons in a lesion of the CNS.

## Additional files

Additional file 1:
**Video 1.** Reversal of the polarity of electric fields (EFs) reversed cell migration direction. Additional file 2:
**Video 2.** Random migration of ARPC2^+/+^ cells without electric field (EF) stimulation. Additional file 3:
**Video 3.** Random migration of ARPC2^−/−^ cells without electric field (EF) stimulation. Additional file 4:
**Video 4.** ARPC2^+/+^ cells migrated toward cathode in electric fields (EFs). Additional file 5:
**Video 5.** Random migration of ARPC2^−/−^ cells in electric fields (EFs).

## Abbreviations

ARP2/3: actin-related proteins 2 and 3
bFGF: basic fibroblast growth factor
BM-MSC: bone marrow mesenchymal stromal cell
BSA: bovine serum albumin
CNS: central nervous system
CQ: cathode-facing quadrant
DC: direct current
EF: electric field
ESC: embryonic stem cell
hESC: human embryonic stem cell
iPSC: induced pluripotent stem cell
NAC: N-acetylcysteine
NCM: neural culture medium
NSC: neural stem cell
NSPC: neuronal stem/progenitor cell
OPC: oligodendrocyte precursor cell
PDGFRα: platelet-derived growth factor receptor alpha
ROCK: Rho-kinase
SCAR: cyclic-AMP receptor
T3: Tri-iodothyronine
WASP: Wiskott-Aldrich syndrome protein
WAVE: Wiskott-Aldrich syndrome protein and verprolin
